# Steerable current-driven emission of spin waves in magnetic vortex pairs

**DOI:** 10.1126/sciadv.ado8635

**Published:** 2024-09-25

**Authors:** Sabri Koraltan, Katrin Schultheiss, Florian Bruckner, Markus Weigand, Claas Abert, Dieter Suess, Sebastian Wintz

**Affiliations:** ^1^ Faculty of Physics, University of Vienna, Kolingasse 14-16, A-1090 Vienna, Austria.; ^2^ Research Platform MMM Mathematics-Magnetism-Materials, University of Vienna, A-1090 Vienna, Austria.; ^3^ Vienna Doctoral School in Physics, University of Vienna, A-1090 Vienna, Austria.; ^4^ Institute of Ion Beam Physics and Materials Research, Helmholtz-Zentrum Dresden-Rossendorf, 01328 Dresden, Germany.; ^5^ Institut für Nanospektroskopie, Helmholtz-Zentrum Berlin für Materialien und Energie GmbH, 12489 Berlin, Germany.; ^6^ Max Planck Institute for Intelligent Systems, 70569 Stuttgart, Germany.

## Abstract

The efficient excitation of spin waves is a key challenge in the realization of magnonic devices. We demonstrate current-driven generation of spin waves in antiferromagnetically coupled magnetic vortices. We use time-resolved x-ray microscopy to directly image the emission of spin waves upon the application of alternating currents flowing directly through the magnetic stack. Micromagnetic simulations allow us to identify the current-driven Oersted field as the main origin of excitation, in contrast to spin-transfer torques. In our case, these internal Oersted fields have an orders of magnitude higher spin-wave excitation efficiency than commonly used stripline antennas. For magnetostrictive materials, we furthermore demonstrate that the direction of magnon propagation can be steered by increasing the excitation amplitude, which modifies the underlying magnetization profile through an additional anisotropy. The demonstrated methods allow for the efficient and tunable excitation of spin waves, marking a substantial advance concerning the design of magnonic devices.

## Teaser

Steerable spin waves generated efficiently by alternating currents in a magnetic stack mark an advance toward magnonic devices.

## INTRODUCTION

State-of-the-art data processing is nowadays based on complementary metal-oxide semiconductor (CMOS) technology, where the control of current flow in transistors is used for logic operations and routing of information (*1*, *2*). Accompanied by memory units such as random access memory for temporary purposes or flash memory for persistent data storage, they build the backbone of today’s computer designs. However, the relatively high power consumption of CMOS devices, due to ohmic losses, volatile refresh memories, and limits for further miniaturization, represents severe challenges for developing a sustainable information and communication technology in the future (*3*).

Magnonic devices have emerged as a candidate solution to the challenges that CMOS is facing today (*4*, *5*). Within such devices, one makes use of magnons (the quanta of spin waves) (*6*) to transfer low-loss information. As a local deflection of the magnetic orientation, spin waves typically exhibit wavelengths from subnanometers to centimeters at frequencies ranging from the megahertz to the terahertz range (*5*, *7*). On the basis of these properties and their intrinsically nonlinear dynamics and coupling mechanisms, they can be used to operate various kinds of devices such as reprogrammable magnonic crystals (*8*), magnonic directional couplers (*9*), Boolean computing devices (*10*), magnonic nanoring resonators (*11*), magnon memory devices (*12*), magnonic logic circuits (*13*), offset-free magnetic field sensor (*14*), spin-wave majority gates (*15*), or even multipurpose devices (*16*–*18*) and many more (*19*, *20*). For real-world implementation of most proposed magnonic applications, it will be crucial to use spin waves of nanoscale wavelengths. This is mainly for two reasons: (i) miniaturization, where the wavelength imposes a constraint on the device footprint, and (ii) high group velocities in the short-wavelength (exchange-dominated) regime for sufficient processing speeds and propagation lengths.

Typically, the excitation of coherent spin waves is achieved by using lithographically patterned microwave antennas, for example, metallic coplanar waveguides (*21*). However, this method has limited efficiency, in particular for nanoscale wavelengths. Here, the wavelengths are restricted on the lower end to the minimum patterning sizes involved and, at the same time, such antennas still need to allow for sufficient impedance matching of the electric circuitry. One way to overcome this limitation, among others, is to couple a global alternating external magnetic field to local internal demagnetization fields (*22*–*24*) or to the dynamics of confined spin textures such as vortex cores (*25*–*30*), skyrmions (*31*–*34*), or domain walls (*35*–*42*). The latter coupling to spin textures was found to be particularly resourceful in synthetic ferrimagnets (SFi’s), where two ferromagnetic layers are coupled antiparallelly through a nonferromagnetic interlayer (*25*, *43*–*45*). On the other hand, it was previously reported that oscillatory dynamics of spin textures can also be excited using the effects from electric currents flowing through these textures themselves, as a result of Oersted fields, spin-transfer torques (STTs), or spin-orbit torques (*46*–*48*). This can be the case for both direct current (dc) and alternating current (ac) as well as for lateral and vertical flow directions.

Thus, it has been a key question, in both fundamental and practical respects, if electric currents flowing through spin textures directly can be used to generate short-wavelength spin waves and how efficient and versatile such a process would be as compared to the global field approach. Here, we demonstrate the realization of such current-driven spin-wave emission in SFi vortex pairs in a lateral ac geometry and without the need for any magnetic bias field. We use high-resolution time-resolved x-ray microscopy to directly image the resulting spin-wave dynamics. Large-scale micromagnetic simulations allow us to understand the origin of the spin-wave emission, distinguish between different excitation mechanisms, and compare their efficiency. When the SFi consists of magnetostrictive materials, we demonstrate that the emission direction of the generated spin waves can be steered by the magnitude of the applied current.

## RESULTS

### Current-driven spin-wave generation

**Fig. 1.**
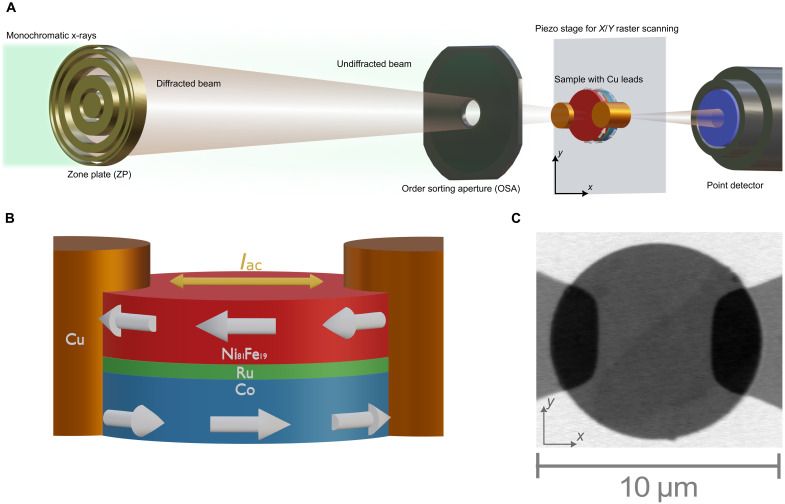
Schematics of experimental setup and SFi sample #1. (**A**) STXM setup, where the monochromatic x-rays are diffracted by the zone plate to focus in a single spot. The combination of the zone plate center stop and the order sorting aperture blocks the undiffracted beam and higher diffraction orders (latter not shown) from reaching the sample. The sample itself is grown on a thin membrane and mounted on a piezo stage that is raster scanned (*x*/*y*) through the focal point. The transmitted x-rays are collected by a point detector. (**B**) Schematic of the Co/Ru/Ni_81_Fe_19_ microdisk with adjacent, partially overlapping copper leads (aspect ratios not to scale). White arrows indicate the general antiparallel orientation of the two ferromagnetic layers of the SFi. Ac injected from the leads flow laterally through the disk as indicated by the orange arrow. (**C**) Static STXM image of the sample recorded at the Fe L_3_ edge. The SFi microdisk (9 μm in diameter) and the copper leads appear as gray contrast areas, with darker contrast in the overlapping regions. Within the disk, there is already faint magnetic contrast visible as well.

For our study, we require a time-resolved magnetic probe beyond the spatial resolution limit of visible-light techniques. Therefore, we image our samples using scanning transmission x-ray microscopy (STXM) with an ~25-nm lateral resolution and by exploiting the x-ray magnetic circular dichroism (XMCD) (*49*) effect as an element-specific magnetic contrast mechanism (see Materials and Methods). The typical STXM measurement setup is illustrated schematically in Fig. 1A. Measurements were carried out at the Maxymus end station (*50*) at the BESSYII electron storage ring operated by the Helmholtz-Zentrum Berlin für Materialien und Energie. The first sample we examine is a circular thin film disk of 9 μm in diameter made out of a Co(47.8nm)/Ru(0.8nm)/Ni_81_Fe_19_(42.8nm) SFi stack (sample #1), as shown schematically in Fig. 1B. Here, we also highlight the electric contacting to the sample, which is provided via copper thin film leads, overlapping at about 2 μm on either side with the microdisk. Note that we use an SFi to image each magnetic layer individually by exploiting the element-specific XMCD effect as well as for being able to modify the magnetic state via the remaining net magnetic moment. A static STXM image of sample #1 recorded at the Fe L_3_ absorption edge is given in Fig. 1C. While the substrate background appears as bright contrast (high photon transmission), both the SFi microdisk and the copper leads appear as gray (medium transmission) or even dark gray where they overlap (low transmission). There is also already a hint for additional magnetic contrast within the disk, which we will turn to in the following.

**Fig. 2.**
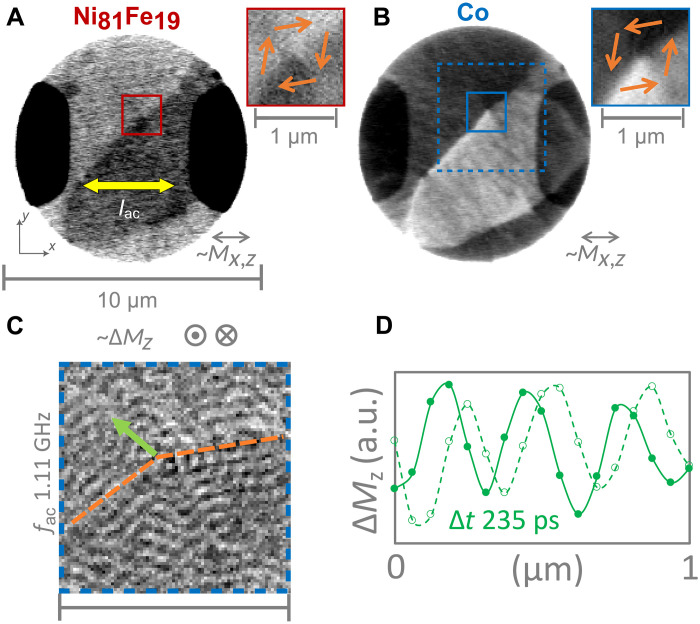
STXM imaging of current-driven spin-wave emission in SFi sample #1. (**A** and **B**) Static STXM images with partial in-plane magnetic sensitivity (∼*M_x_*), recorded at the Fe L_3_ edge (A) and the Co L_3_ edge (B). Zoom-in panels on the right correspond to the solid squares in (A) and (B), with orange arrows indicating the general magnetic vortex pair orientations as given by the black and white contrast. (**C**) Normalized TR-STXM snapshot of spin-wave dynamics in response to an ac of *f*_ac_ = 1.1 GHz flowing laterally through the disk, recorded with perpendicular magnetic sensitivity (∼Δ*M_z_*) at the Co L_3_ edge. (**D**) Spin-wave amplitude profile extracted along the green arrow in (C) for a relative time delay of Δ*t* = 235 ps. a.u., arbitrary units.

To identify the magnetic state of sample #1, we performed static STXM imaging with partial in-plane magnetic sensitivity, separately for the two ferromagnetic layers. In addition to topographic information, Fig. 2 (A and B) reveals the horizontal projection (*M_x_*) of the magnetic orientation for the Ni_81_Fe_19_ layer (recorded at Fe L_3_) and the Co layer (recorded at Co L_3_), respectively. By means of these images, it becomes obvious that the in-plane magnetic components of the two layers are coupled antiparallelly (opposite relative contrast) and that there is a multivortex-domain state within the microdisk (multiple vortex cores in each layer). Despite this multidomain state, a large portion of the disk area can be identified as being part of a single interlayer vortex pair with antiparallel vortex circulations that have a slightly discrete and distorted rotation character (wall formation with rotation angles above and below 90°). The corresponding vortex cores are being somewhat displaced congruently to the upper right from the center of the disk. Zoom-in panels of the center regions (red and blue solid frames) allow for a closer view of the core area, with orange arrows illustrating the general vortex pattern. Although the core polarity cannot be directly obtained from these particular two images, it was found that, typically, the cores are aligned parallel in this kind of system (cf. fig. S1) (*25*). Note that, in general, the STXM images of the Co layer have a higher signal-to-noise ratio than those of the Ni_81_Fe_19_ layer as there are many more absorbing Co atoms than Fe atoms in the sample, at a similar level of XMCD.

To evaluate if current-driven spin-wave emission is possible in this kind of system, we send an ac laterally through sample #1, which is injected via the adjacent copper leads. The microwave current had a frequency of *f*_ac_ = 1.11 GHz and a relevant current density amplitude of *j* = 2.9 × 10^10^ A m^−2^ and is flowing mainly along the *x* axis, connecting the leads. Note that, for the calculation of the current density, we assume a uniform current flow in the SFi stack, where the cross-sectional area is defined as *A* = *l* × *d*, where *l* is the diameter of the disks (*l* = 9 μm) and *d* is the total thickness. Using time-resolved STXM (TR-STXM) (see Materials and Methods), we directly observe the dynamic response of the system. Figure 2C shows a TR-STXM normalized snapshot in time Δ*M_z_*(*t*_0_), being sensitive to dynamic changes of the perpendicular component *M_z_* of the magnetization of the Co layer. The area represented by the blue dashed box in Fig. 2B is the corresponding image area. We observe a clear spin-wave pattern of substantial amplitude with an average wavelength of 307 ± 30 nm. By looking at consecutive snapshots in an animated way (movie S1), it becomes clear that these waves are mainly emitted from the somewhat bend, higher-angle domain walls (highlighted by orange dashed lines in Fig. 2C) and the vortex core. Note that the background magnetic state in Fig. 2C is different from that in Fig. 2 (A or B) with respect to the state of the domain wall (sample was remounted in between). The emission pattern can be seen in further detail in fig. S1, which displays the dynamic snapshots for the two layers separately in both absolute and normalized visualization, also revealing an in-phase dynamics of the *M_z_* component of the two layers. Thereby, we can conclude that, in line with earlier reports (*25*, *44*), the observed spin waves are an instance of the acoustic, layer-collective SFi mode in the Damon-Eshbach geometry. Figure 2D further highlights the propagating nature of the observed spin waves by plotting the amplitude profile along the green arrow in Fig. 2C for a relative time delay of 235 ps and with additional lines for guiding the eye. The phase propagation is clearly visible. Note that the observed excitation process is not restricted to a certain frequency or resonance but rather covers a broadband frequency range from hundreds of megahertz to multiple gigahertz (*42*) as shown exemplary in fig. S2 and movie S2. Our experiments evidently demonstrate that short-wavelength spin-wave emission in spin textures can be driven by ac’s flowing through these textures.

### Origin of the current-driven emission

In the following, we investigate the origin of the spin-wave emission from spin textures by using micromagnetic simulations with magnum.np (*51*) (see Materials and Methods for more details). When a charge current flows directly through a ferromagnetic material, several effects may occur to back-act on its magnetic state; two of which are the most prominent for our case. On the one hand, the charge current flowing along a given direction will generate an Oersted field within the conductor that is often axially symmetric. On the other hand, electrons that have acquired a spin polarization may be exerting a torque on the magnetization, termed the STT (*52*, *53*). Both effects can be described by means of a micromagnetic continuum model (*54*). Concerning the STT effect, note that electrons performing a lateral motion in a lateral system can exert a torque on the magnetization for the case that there is local magnetization gradient. Although, in reality, they are occurring at the same time, we can use micromagnetic simulations to apply them together, or individually, to investigate their role on the emission of spin waves. Thus, we perform such micromagnetic simulations, numerically solving the Landau-Lifshitz-Gilbert (LLG) equation to understand the origin of the spin-wave emission that we observed experimentally. Note that we chose a system analogous to sample #1 but limited the lateral size of the simulated disk to 4 μm for computational practicability reasons. Nevertheless, we confirmed that very similar results arise in even smaller disks, which validates our approach. Noteworthy, it is crucial to use double precision floating point operations to perform the numerical calculations to achieve the necessary accuracy.

**Fig. 3.**
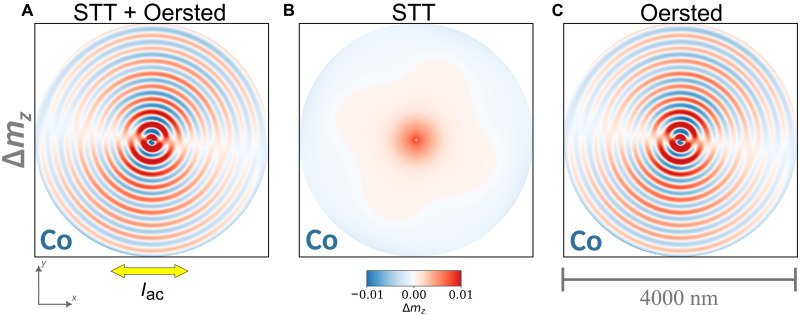
Origin of current-driven spin-wave emission in SFi vortex pairs (simulations). Snapshots of the normalized perpendicular dynamic magnetization (Δ*m_z_*) in the Co layer (averaged over the layer thickness) in a system analogous to sample #1 obtained from micromagnetic simulations with the contrast according to the blue-white-red color bar. The current density was modeled to *j* = 1 × 10^9^ A m^−2^ at *f*_ac_ = 1.11 GHz. In (**A**), both the STT and the current-driven Oersted field were included in the effective field, while in (**B**) and (**C**), only the STT and the Oersted field were considered, respectively.

Figure 3 summarizes the different excitation methods simulated for an ideal vortex pair state with opposite circulations and parallel cores. It allows us to identify, in general, the main contributor to the current-driven emission of spin waves in our case. Figure 3A depicts the steady-state magnetization profile in the Co layer during the application of an ac density of *j* = 1 × 10^9^ A m^−2^ and *f*_ac_ = 1.11 GHz, where contributions from both the STT and the Oersted field are included in the modeling. For the sake of simplicity, we assume a uniform current flow along the *x* axis throughout the stack. In reality, position-dependent areal cross sections, different resistivities of the materials, and misalignments of the copper leads might induce inhomogeneities. To test the validity of this assumption, we have performed finite-element simulations using the COMSOL software, where a static Ohm solver is used to calculate the vector of the current flow based on a realistic modeling of the lateral contacts and different resistivities (see fig. S11). We found that the current density and direction is nearly homogeneous within a width of ~5 μm but starts to deviate and curve toward the edges and closer to the lateral contacts. This given and taking into account that the skin effect is negligible at the given frequencies and film thicknesses, we can justify our assumption of a homogeneous current density in our micromagnetic simulations.

One can see the emission of coherent, high-amplitude spin waves with wavelengths similar to those in the experiment. In contrast, there is no emission of spin waves anymore if one takes into consideration only the STT as shown in Fig. 3B. Although the STT acts locally wherever the magnetization profile has a spatial gradient, the amplitude of the applied current is not sufficient to excite the magnetic vortex cores to gyrate to emit spin waves. If one considers only the generated Oersted field, then the resulting spin-wave pattern can be again observed in Fig. 3C, which is almost identical to the one in Fig. 3A. Therefore, we can conclude that it is solely the Oersted field that causes the efficient excitation of spin waves in our experiment. The magnetization dynamics only driven by Oersted fields is shown in movie S3, and movie S4 contains animations of spin-wave emissions for different excitation methods. In addition, we provide the results from simulations with excitation current densities of the same order as in the experiment in fig. S3, where the magnetization and the normalized perpendicular dynamic magnetization are displayed separately for the Co layer and the Ni_81_Fe_19_ layer. These simulations demonstrate that the spin-wave amplitude increases with the applied current density, while we also observe a noticeable double-frequency (2*f*) excitation of spin waves at the same time. The current-induced excitation of spin waves is a broadband technique for frequencies ranging from hundreds of megahertz to several gigahertz, as demonstrated for sample #1 in fig. S12. The dispersion relation *f*(*k*) was obtained after performing individual micromagnetic simulations for each of the frequencies, and it agrees reasonably well with the experimental data points and the results obtained by a dynamic matrix approach in ref. (*55*). The dispersion curve exhibits a slight parabolic increase in frequency with wave number, resulting in a group velocity (2πd*f*/d*k*) that linearly increases with *k*, at an average value of ~600 m/s. It is noteworthy that the simulated spin-wave excitation efficiency drops monotonously for increasing frequencies up to 5 GHz and remains at a relatively low level up to 10 GHz. In addition to this predicted intrinsic spin-wave amplitude drop, there may be technical effects from a reduced microwave transmission at higher frequencies, which could be partially compensated for by optimizing the impedance matching of the circuit.

It is worth noting that the excitation could be achieved also by using a compensated synthetic antiferromagnet (SAF), e.g., using two antiferromagnetically coupled NiFe layers. For the sake of completeness, we provide micromagnetic simulations in fig. S9 where we demonstrate that a compensated SAF can be excited with the same method as well.

### Excitation efficiency

As we identified the Oersted field as the origin of the current-driven spin-wave emission, we further evaluate the excitation efficiency of this method. To this end, we compare the current-driven Oersted field–dominated spin-wave emission to the previously used stripline antenna–driven spin-wave emission from vortex cores (*25*, *42*) and also to the STT excitation above yet considering two orders of magnitude higher current densities.

**Fig. 4.**
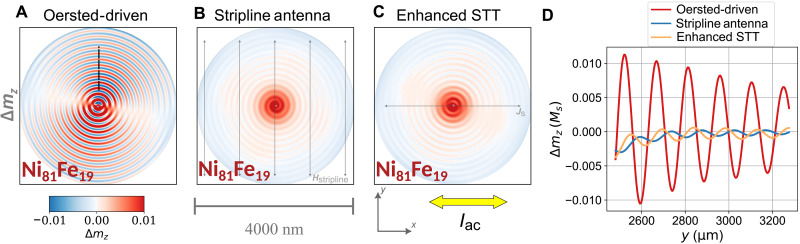
Comparison of the spin-wave excitation efficiency in SFi vortex pairs (simulations). Averaged spin-wave amplitude maps of the Ni_81_Fe_19_ layer, given in terms of Δ*m_z_* snapshots during excitation with (**A**) current-driven Oersted field, (**B**) uniform field of equal magnitude $\text{max}\left(H_{y}^{\text{Oersted}}\right)=H_{y}^{\text{uniform}}=41.8\;A\;m^{-1}$ (as if the magnetic field was generated by a stripline antenna), and (**C**) enhanced STT excitation with a 100-fold increased current density. The excitation frequency is *f* = 2 GHz. The spin-wave amplitude along the black dotted line depicted in (A) is plotted in (**D**) for the three different cases according to the color code in the legend in (D).

Figure 4 gives an overview of the spin-wave amplitude profiles obtained from our micromagnetic simulations, this time at a frequency of *f*_ac_ = 2 GHz. The current amplitude is again *j*_ac_ = 1 × 10^9^ A m^−2^. In contrast to Fig. 3, we now depict the magnetization of the Ni_81_Fe_19_ layer, yet as shown above, the Co layer behaves equivalently in terms of the perpendicular spin-wave component. The color-coded change in the normalized dynamic Δ*m_z_*, component of the magnetization, this time averaged over the Ni_81_Fe_19_ layer, is strongest if the spin waves are excited via the current-driven Oersted field method, as can be seen in Fig. 4A. As referred to above, two other cases of spin-wave excitation were modeled numerically. The first involves a stripline antenna situated underneath the vortex pair, which produces an Oersted field that acts on the SFi (Fig. 4B). The second case involves only the STT from the current flowing through the sample yet increasing the injected current density by a factor of 100 (Fig. 4C). Both of these methods can excite spin waves, but the current-driven Oersted field generates spin waves with much higher amplitudes, making it the most effective excitation method of the three cases discussed (see also movie S4). The excitation efficiency can be further highlighted by means of the amplitude profiles in Fig. 4D. Note that the stripline antenna is assumed to generate a uniform field of equal magnitude compared to the current-driven Oersted field $H_{y}^{\text{uniform}}=\text{max}\left(H_{y}^{\text{Oersted}}\right)=41.8\;A\;m^{-1}$. Comparing the spin-wave excitation efficiency of the three methods, we see that the amplitude of the spin waves that were excited by current-driven excitation is a factor of ~10 higher compared to the enhanced STT excitation and a factor of ~30 higher compared to the excitation with the stripline antenna. In terms of energy coupling efficiency (considering the square of the amplitude), it means that current-driven Oersted field excitation has an impressive benefit of three orders of magnitude for the case of the given system as both types of devices have typically a similar dc resistance/power loss.

### Mechanisms of excitation

**Fig. 5.**
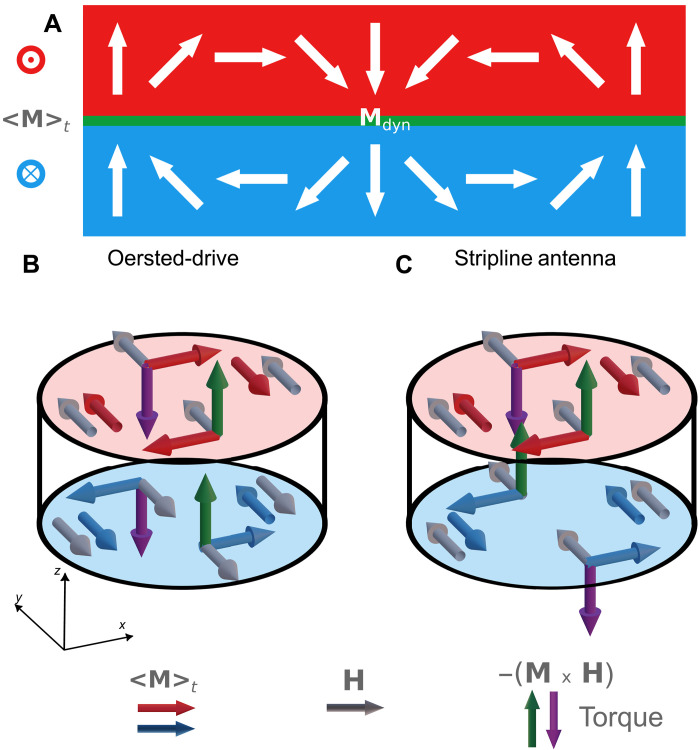
SFi spin-wave profile and excitation symmetries (schematics). (**A**) Cross-sectional profile of the dynamic magnetization components (white arrows) of an acoustic Damon-Eshbach spin wave in the SFi system with colors (blue and red). Note that, here, acoustic means that the perpendicular dynamic components are in-phase (in contrast to the dynamic in-plane components, which are antiphased). (**B** and **C**) Equilibrium magnetization in the antiparallel SFi vortex pairs (blue and red arrows) in the respective layers (blue and red disks) and Oersted fields (gray arrows) at a snapshot in time for the Oersted-driven case (B) and stripline-antenna case (C). The locally resulting torque (−**M** × **H**) is depicted by green (up) and purple (down) arrows. From a symmetry perspective, only the Oersted-driven excitation can directly excite the acoustic Damon-Eshbach SFi mode as, here, the *z* torques are in-phase between the two layers (B), in contrast to the stripline antenna excitation, where they are antiphased (C).

The reason why the spin-wave generation efficiency is much higher for the current-driven case than for the uniform (stripline) case presumably lies in the symmetry of the excited spin-wave mode. This mode is the acoustic collective Damon-Eshbach mode of the SFi, which has in-phase perpendicular dynamic components and antiphase in-plane dynamic components for the two ferromagnetic magnetic layers (Fig. 5A) (*25*). Because the two layers have antiparallel in-plane equilibrium magnetic orientations, this mode cannot be directly excited by means of uniform in-plane fields as a consequence of symmetry considerations (see Fig. 5C) (yet it could be excited for perpendicular uniform fields). For the Oersted field, however, the actual field orientations acting on the two ferromagnetic layers are opposite (see Fig. 5B), and therefore, a direct excitation of the acoustic mode is possible from symmetry arguments. Note that the actual Oersted field distribution is inhomogeneous as shown in fig. S15, yet its general in-plane symmetry remains. The above considerations are valid for the quasiuniform precession case, but not necessarily for the excitation of short-wavelength spin waves. In the uniform in-plane field case (and the enhanced STT case), wave excitation requires the presence and gyration dynamics of the vortex cores, that can be driven by in-plane fields and then act as kind of a perpendicular nanostirrer, leading to a spiraling wave pattern (*25*, *42*). For the Oersted field case, the situation is different. Here, the vortex cores are not strictly required for the excitation of waves. Simulations with artificially removed cores show comparable results to those conducted with the cores present, as shown in fig. S8. Consequentially, there is no spiraling signature in the resulting emission pattern, typically originating from vortex core gyration. The reason why nevertheless a finite wavelength is excited, lies in the very strong dispersion nonreciprocity of the acoustic Damon-Eshbach mode in the SFi (*25*, *45*). Here, nonreciprocity refers to the fact that counterpropagating waves of the same frequency have significantly different wavelengths (by a factor of the order of 10). Therefore, the global Oersted field can excite a quasihomogeneous precession of the order of half the structure size (radially), and upon lateral radial reflection, this dynamics appears as a short-wavelength branch propagating away from the cores (or the rim for the other antiparallel relative vortex circulation combination possible). We refer to this effect as nonreciprocal excitation. Note that, in the simulations, there is also a dependence of the spin-wave amplitude on the azimuthal angle, exhibiting a twofold symmetry. In particular, there appears a quasinodal line, crossing the disk center from left to right. This effect stems from the azimuth-dependent resulting torque of the form ∝(−**M** × **H**^Oersted^), when considering the Oersted field geometry and the vortex magnetization. Figure 5B illustrates the torque generated by the Oersted field, and Fig. 5C depicts the torque resulting from the uniform external field from a stripline antenna. The possible extent to which the spin-texture dynamics (vortex core gyration and domain wall oscillation) still contributes to the excitation of waves in parallel to the nonreciprocal Oersted field excitation effect remains a challenge to be quantified.

### Direction-steerable spin-wave emission

In the above experiments and simulations, the spin-wave emission pattern was fixed by the specific geometries of the spin texture and the current flow. In the following, we will demonstrate a proof-of-principle system where the emission pattern and, in particular, its directional properties can be tuned by the drive current. To this end, we used another SFi disk of the same diameter (9 μm) made of a Ni_81_Fe_19_(44.9 nm)/Ru(0.8 nm)/Co_40_Fe_40_B_20_(46.6 nm) stack (sample #2), which is illustrated in fig. S4. Co_40_Fe_40_B_20_ is well known to be a magnetostrictive material (*56*, *57*), i.e., it changes its anisotropy with mechanical strain through the magnetoelastic effect.

**Fig. 6.**
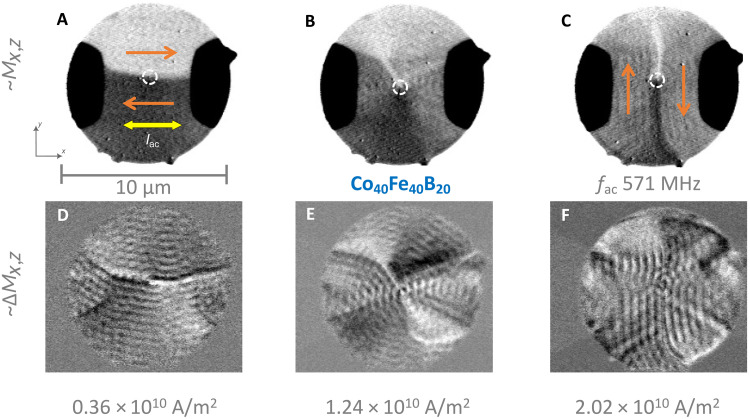
TR-STXM imaging of direction-steerable spin-wave emission in SFi sample #2. Response to an ac of *f*_ac_ = 571 MHz with current densities as provided below the individual columns. Images recorded at the Co L_3_ edge. (**A** to **C**) Direct absorption snapshots showing both topographic and magnetic contrast, the latter with mixed sensitivity (∼*M*_*x*,*z*_). White dashed lines indicate the position of the vortex cores. (**D** to **F**) Normalized snapshots that highlight the magnetic dynamics (∼Δ*M*_*x*,*z*_). The orange arrows indicate the magnetic background orientation, and the yellow arrow indicates the current direction.

Figure 6A shows an STXM image with partial in-plane sensitivity of the Co_40_Fe_40_B_20_ layer of sample #2. Although excited by an ac, the overall magnetic state is analogous to the equilibrium state of the sample. As a consequence of an intrinsic uniaxial magnetic anisotropy along the *x* axis (horizontal), the vortex circulation is broken up into two oppositely oriented domains as indicated by the orange arrows. These domains are connected by 180° domain walls that have a vortex core remaining in the center (*42*). The origin of the intrinsic uniaxial anisotropy presumably stems either directly from the film growth or from a potential tensile strain exerted by the copper leads, as also found in an earlier study (*42*). In particular, it was reported that both inclined sputtering and deposition under magnetic fields can induce uniaxial in-plane anisotropies in Co-Fe-B compounds (*58*, *59*). As for sample #1, the Ru-mediated interlayer exchange coupling enforces an antiparallel alignment of the magnetic in-plane components between the two ferromagnetic layers of sample #2, as can be seen in fig. S4.

Figure 6A shows the response of the sample as TR-STXM snapshots with mixed in-plane and out-of-plane sensitivity (∼*M*_*x*,*z*_) toward an ac of *f*_ac_ = 571 MHz flowing through the sample at *j* = 0.36 × 10^10^ A m^−2^ amplitude. It shows the direct absorption view, where magnetic and nonmagnetic features are visible (the disk itself, the copper leads, the magnetic domains, and some signature of spin waves). Figure 6D shows the corresponding snapshot as normalized view, with sensitivity to magnetic changes over time compared to the time-average state (∼Δ*M*_*x*,*z*_). In this view, we see a clear emission pattern of rather plane spin waves from the horizontal domain walls into both directions (up and down) with a wavelength of (472 ± 15) nm. There are also oscillations of the domain wall itself visible with some additional complexity in the regions outside the center area, below the leads (possible wall branching). For the animated version of Fig. 6, we refer to movie S5. When the current density is increased to *j* = 1.24 × 10^10^ A m^−2^, we see in Fig. 6 (B and E) that the time-average state has changed compared to the low-current/static state. The earlier well-defined horizontal domain walls have changed into a more continuous vortex state, with some diagonal wall features remaining. As one can see in the normalized snapshot in Fig. 6E, the spin-wave emission pattern is now more isotropic than before with wavelengths of (481 ± 15) nm. When the current density increases further to *j* = 2.02 × 10^10^ A m^−2^, the time-averaged state changes again, as shown in Fig. 6 (C and F). There is a reappearance of well-defined 180° domain walls, however, this time they are oriented along the *y* axis (vertical). The resulting spin-wave emission pattern in Fig. 6F mainly consists of plane waves again emitted from the domain walls, now propagating along the horizontal axis with a wavelength of (516 ± 15) nm. By tuning the excitation current density, we can control the orientation of the domain walls in sample #2 to be horizontal or vertical, which in turn gives us control of the axis along which plane spin waves are emitted. For intermediate current densities, the system assumes a less anisotropic state, with almost isotropic spin-wave emission. At the same time, the spin-wave wavelength appears to be almost unaffected by this way of steering the emission direction.

The most likely explanation for the reorientation of the domain walls lies in a combination of Joule heating and magnetoelastic effects (inverse magnetostriction). With increasing current density, the disk starts to warm up, urging it to expand. Here, the copper leads provide a certain constraint for a horizontal expansion, which exerts essentially a compressive strain on the disk. In the course of this action, the initially horizontal anisotropy is first reduced and eventually realigned to a vertical orientation. This explanation is in line with the positive magnetostrictive constant of Co_40_Fe_40_B_20_ (*60*, *61*), while the magnetosrictive effects in Ni_81_Fe_19_ are negligible (*62*). It is also consistent with the fact that the anisotropy control is rather independent of the driving frequency, as can be seen in fig. S5 and movie S6, showing similar state responses at *f*_ac_ = 71 MHz and *f*_ac_ = 1071 MHz. Furthermore, it was already reported in the literature that an easy magnetic axis can be transformed into a hard axis when Co_40_Fe_40_B_20_ is strained (*63*). Because we observe a reorientation of anisotropy instead of the occurrence of a fourfold anisotropy, we conclude that the intrinsic as-grown/as-patterned anisotropy is largely related to strain.

To check the validity of our assumptions, we perform COMSOL simulations, which are summarized in fig. S11. Here, we solve the static equations for electromagnetic heating and the resulting mechanical deformations. We can confirm that, for a magnetostrictive material such as CoFeB, the disks are deforming in such a way that they induce a strain along the *y* axis, perpendicular to the direction of the current. Ultimately, we validated that the strain-induced anisotropy might be induced by the Joule heating present in the system. Note that the chosen constraints, such as fixed mechanical constraints and toward which direction the heats dissipates, might change the magnitude of the Joule heating and total displacement accordingly. Further details can be found in the Supplementary Materials.

**Fig. 7.**
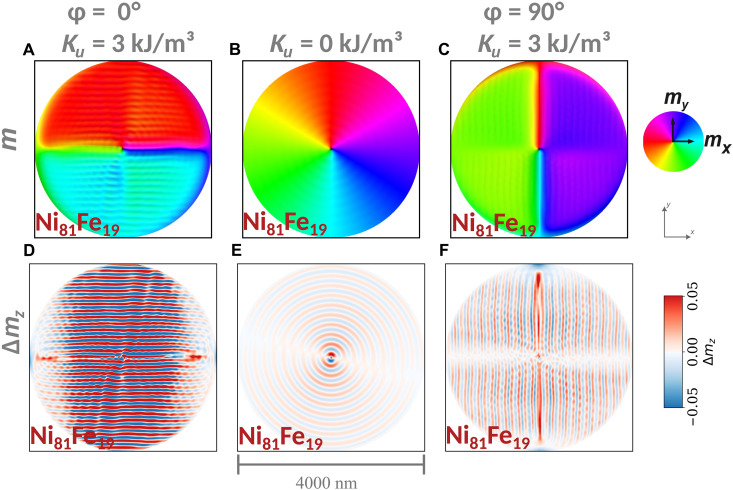
Direction-steerable spin-wave emission in anisotropic SFi vortex pairs. (**A**) Magnetization of the bottom Ni_81_Fe_19_ layer as a color-coded snapshot, where the horizontal in-plane domains with the vortex core are visible. The contrast displays all magnetization components including the modulation of magnetization by the spin-wave dynamics. The purely dynamical out-of-plane component of the magnetization, equivalent to the spin-wave amplitude, is highlighted in (**D**) via Δ*m_z_*. In (A) and (D), the strain-induced uniaxial anisotropy in the Co_40_Fe_40_B_20_ layer was set to be parallel to the applied current (φ = 0) with an anisotropy constant of *K_u_* = 3 kJ/m^3^. This anisotropy was set to zero in (**B**) and (**E**) to simulate the current/strain-induced compensation of the anisotropy. To simulate the rotation of the anisotropy, leading to the emission of spin waves in the vertical direction, the anisotropy axis was rotated to align along the *y* axis (φ = 90°) in (**C**) and (**F**).

We underpin this hypothesis by further micromagnetic simulations, where we model sample #2 with dimensions equal to those used for sample #1. To this end, first, we introduce an in-plane anisotropy to the effective field along the *x* axis. We demonstrate in fig. S7 that, by increasing the anisotropy constant *K_u_*, a bidomain state is induced, similar to that observed in our experimental findings. Then, we simulate the current-driven Oersted field excitation to model the corresponding spin-wave generation. In Fig. 7, we depict three cases. First, the anisotropy axis is chosen to be along the *x* axis and parallel to the applied current (Fig. 7, A and D), where *K_u_* = 3 kJ/m^3^. The strain-induced magnetoelastic anisotropy can be expressed as $K_{\text{ME}}=\frac{3}{2}\lambda_{s}\epsilon E_{F}$ , where λ_s_ is the saturation magnetostrictive constant, ϵ is the strain, and *E_F_* is the Young’s modulus. Assuming that the strain is given by the ratio of the average displacement and total length of the disks as obtained by the COMSOL simulations for *I* = 16 mA, i.e., ϵ = 0.056%, and λ_s_ = 31 ppm (parts per million) and *E_F_* = 160 GPa from the literature (*63*), we can approximate a magnetoelastic anisotropy constant *K*_ME_ = 4.2 kJ/m^3^ for Co_40_Fe_40_B_20_, which is within the same order of magnitude as we have assumed in our micromagnetic simulations.

Figure 7D shows a clear anisotropic emission of plane spin waves, originating at the domain wall. By that, we reproduce the low current density regime in Fig. 6D. If we assume that, upon the intermediate increase in the current density, the resulting magnetoelastic effect compensates for the original anisotropy, then a regular vortex pair is obtained again as shown in Fig. 7B, where the spin-wave emission is now rather isotropic, as can be seen in Fig. 7E. Last, when the magnetoelastic effect induces the domain wall to rotate by 90°, the emission of spin waves occurs colinear to the current direction (Fig. 7, C and F). We provide the animations analogous to Fig. 7 in movie S7.

Similar as for sample #1, the spin-wave generation and steering methods presented for sample #2 work in a broad range of frequencies. In figs. S13 and S14, we provide the simulated dispersion relations for the two orthogonal orientations of propagation. In addition, we demonstrate in fig. S10 that steering can also be achieved in smaller disks of 1, 2, and 3 μm in diameter for which we repeated the micromagnetic simulations. However, we also observe that, for smaller diameters, the steering effect gradually reduces as a consequence of a growing influence of demagnetization effects over the size-independent intrinsic anisotropies.

Furthermore, the steering experiments were performed repeatedly, where the low- and high-current regimes (and thus different spin-wave emission directions) were investigated in an arbitrary yet repeated order. From the results, we can conclude that the steering process is fully reversible, which means that the direction of the spin-wave emission can be fully controlled by the drive current. In addition, we performed time-dependent COMSOL simulations to understand the timescales required for the Joule heating to stabilize and found that it takes about 400 ns. Hence, the amplitude-induced steering of spin waves can be achieved at submicrosecond scales. Note that, while there are certainly clear benefits in being able to control the spin-wave emission direction by the drive current, there are also other, potentially more practical and faster ways to control strain/anisotropy directly, e.g., by piezoelectrics or voltage-controlled magnetic aniostropy. In addition to direction-steerable emission, sample #2 can also be used to demonstrate single pulse–driven excitation of individual spin-wave wave packages (see fig. S7 and movie S8). This effect further highlights the agility of current-induced spin-wave excitation in the SFi, neither requiring particular resonance frequencies nor multiple onset excitation cycles.

## DISCUSSION

We demonstrated efficient current-driven excitation of spin waves in an antiferromagnetically coupled magnetic vortex pair. By using TR-STXM, we showed that the spin waves are generated when the SFi is subjected to an ac flowing directly through it. Micromagnetic simulations revealed that the Oersted field generated in the SFi by the current is the main contributor to the emission of spin waves in the magnetic vortex pair. By further comparing this to the contributions from both the STT and the uniform magnetic field of a stripline antenna, we determined that the excitation efficiency of the current-driven Oersted field in terms of energy is at least three orders of magnitude higher than the other two methods investigated. We also demonstrated that the presence of magnetic vortex cores is not a necessity anymore for the excitation of short-wavelength spin waves when using current-driven Oersted fields as the long-wavelength branch of the nonreciprocal acoustic Damon-Eshbach mode can be efficiently excited through the symmetry of the field. At a dc resistance of 10 ohms, the present devices exhibit power losses of the order of 10 μW to 1 mW, depending on the particular current density applied. However, when only considering the standard electric resistivities of the materials involved, the achievable device resistance can be much lower, leading to potential ideal power losses between 1 and 100 μW without any additional design optimization. To demonstrate an additional advancement of current-driven spin-wave generation, we prepared the SFi from a magnetostrictive material, where initial strain induces an additional anisotropy. This additional anisotropy distorts the vortex state, leading to the formation of a bidomain state with a pronounced domain wall in between the domains in both magnetic layers. We demonstrated that the propagation direction of plane spin waves excited in this system can be redirected if the amplitude of the applied current is varied, allowing even for a 90° change in the emission direction. Our findings represent a substantial advance in the current-driven, energy-efficient excitation and control of spin waves for potential magnonic nanodevices.

## MATERIALS AND METHODS

### Sample preparation

Analogous to refs. (*25*) and (*42*), the magnetic multilayers were deposited by means of magnetron sputtering onto x-ray transparent silicon nitride window membranes in the presence of an in-plane magnetic field. For oxidation protection, an aluminum capping layer of 3 nm in thickness was used. The microdisks were fabricated by means of electron beam lithography (EBL) and consecutive ion beam etching. After an initial oxygen plasma treatment for adhesive purposes, a negative resist (MA-N 2910) was spun onto the films and baked out. In a second step, the microdisks were exposed by EBL and the samples were then developed for 300 s in MA-D 525 and subsequently rinsed in deionized water (60 s). Last, the samples were milled using an argon ion beam at two different angles (85° and 5°) with endpoint detection for ~1 hour to physically etch the magnetic microdisks out of the continuous films. An acetone bath (12 hours) and a second oxygen plasma step (20 min) were applied to remove the remaining resist. For being able to apply electric currents flowing laterally through the samples, copper/aluminum leads of 200/5 nm in thickness were fabricated to overlap at about 2 μm with a microdisk at two opposing rim positions (see Fig. 1). For this purpose, EBL, electron beam evaporation, and lift-off processing were used. The resulting resistances of the samples were typically between 10 and 100 ohms.

### Scanning transmission X-ray microscopy

To image the magnetic orientation in the multilayer disks, synchrotron-based STXM was used (*50*), as schematically depicted in Fig. 1A. For STXM, a Fresnel zone plate is used to focus monochromatic x-rays onto the sample, while undiffracted x-rays and those of higher diffraction orders are blocked by a combination of the zone plate center stop and a circular order selecting aperture. The x-ray intensity transmitted through the sample is collected by a point detector. To get an image, the sample is raster scanned through the focused beam, yielding a lateral resolution of ~25 nm. Magnetic contrast is achieved by exploiting the XMCD effect (*49*), occurring at the element-specific resonant absorption edges. For the given magnetic multilayer of sample #1, therefore, the two layers can be separately imaged in terms of magnetic orientation by measuring at photon energies of Co L_3_ ∼778 eV and Fe L_3_ ∼708 eV or Ni L_3_ ∼853 eV. For sample #2, on the other hand, Co and Ni photon energies lead to layer-selective images, while the Fe energy provides an integrated signal of both layers. In general, the logarithmic transmission contrast detected is proportional to the projection of the magnetization on the x-ray propagation direction, which means that, in normal incidence, XMCD is sensitive to the perpendicular magnetization component, while inclination of the sample provides additional information on in-plane magnetization components.

The spin-wave dynamics in the multilayer disks was imaged using pump-and-probe TR-STXM (*50*). For TR-STXM, the inherent time structure of the incident x-ray pulses (500-MHz repetition rate and ∼100-ps effective pulse width) is used. The accessible sinusoidal excitation frequencies are of the form *f_M_* = *M*/N*500 MHz, where *M* is an integer multiplier and *N* corresponds to the integer number of phases acquired simultaneously. The level of the excitation signal was measured both before and after the sample (via a pick-off tee) by means of an oscilloscope. The average of the input and transmitted signal was used to estimate the current flowing through the sample by considering a circuit resistance of 50 ohms at the probing oscilloscope. From this current, the current density was estimated by considering the sample cross section transversal to the current (diameter x thickness). We assume a maximum relative uncertainty of about ±25% for the current densities estimated in such way, stemming from losses in the coaxial connects (signal attenuation not considered) and from the fact that a pick-off tee instead of a directional coupler was used to measure the input voltage (reflected signal cannot be isolated).

### Micromagnetic simulations

We use magnum.np (*51*), an open-source graphics processing unit accelerated micromagnetic simulation library based on PyTorch, to conduct our numerical investigations. We modeled the SFi stack in a total simulation box of 1000 × 1000 × 20 cells with discretization lengths (4,4, and 4.5 nm), where the first 10 cells along the dimension *z* are chosen to be the bottom layer and the other 10 cells represent the top layer. For sample #1, we use the following material parameters $M_{s}^{\text{Co}}=1240\;\text{kA}/m$ , *A*^Co^ = 16 pJ/m, and $K_{u}^{\text{Co}}=0$ for the bottom Co layer as well as $M_{s}^{Ni_{81}Fe_{19}}=750\;\text{kA}/m$, $A^{Ni_{81}Fe_{19}}=7.5\;\text{pJ}/m$, and $K_{u}^{Ni_{81}Fe_{19}}=0$ for the top Ni_81_Fe_19_ layer, in agreement with the previous works (*25*, *42*). Each layer is modeled as a disk with a diameter of 4 μm, where cells outside the disk are initialized with *M_s_*, *A*, *K_u_* = 0, as usual in finite difference–based micromagnetic simulations. We use second-order Ruderman-Kittel-Kasuya-Yosida (RKKY) coupling to ensure the correct antiferromagnetic coupling between cells (*64*). The coupling strength is chosen to be *J*_RKKY_ = −0.1 mJ/m^2^. For sample #2, the material parameters were chosen as $M_{s}^{Co_{40}Fe_{40}B_{20}}=1240\;\text{kA}/m$, $A^{Co_{40}Fe_{40}B_{20}}=12\;\text{pJ}/m$, and $K_{u}^{Co_{40}Fe_{40}B_{20}}$ was varied between 0 and 3 kJ/m³ for the top Co_40_Fe_40_B_20_ layer. We used $M_{s}^{Ni_{81}Fe_{19}}=750\;\text{kA}/m$, $A^{Ni_{81}Fe_{19}}=8\;\text{pJ}/m$, and $K_{u}^{Ni_{81}Fe_{19}}=0$ for the bottom Ni_81_Fe_19_ layer. The Gilbert damping is α = 0.01. In all cases, we assume an artificially increased damping region of α = 0.1 for the annulus of the outermost 50 nm. This allows us to avoid reflections of the spin waves at the rim of the sample and thus to achieve the steady state much faster.

We begin our simulations by parametrizing an SFi with two magnetic vortices of opposite circulations but parallel polarities of the vortex cores. We relax the magnetization state by numerically integrating the LLG equation at a high Gilbert damping parameter of α = 1 for 20 ns, where

$$\frac{\partial m}{\partial t}=-\gamma m\times H^{\text{eff}}+\alpha m\times \frac{\partial m}{\partial t}$$ (1)

describes the temporal evolution of the magnetization vector field. Here, γ = 2.2128 × 10^5^ m/(A·s) is the gyromagnetic ratio, and **H**^eff^ is the effective field that can be derived as the variational derivative of the different relevant energetic contributions. For the statical relaxation process of the total energy, the effective field includes only contributions from the demagnetization, exchange, uniaxial anisotropy, and interlayer exchange (RKKY) energies. Note that we do not use a physical spacing layer but a second-order finite-difference implementation of the RKKY (*64*). This will slightly change the stray fields in between the two magnetic layers in comparison to the experiments, but it suffices to reproduce the most relevant effects. Once the magnetization is relaxed, we include additional dynamic contributions such as the current-induced Oersted field, the alternating uniform Zeeman field, or the STT. Note that all terms are already implemented in magnum.np and can be used together or individually as desired.

### Oersted field

The charge current that passes through a conducting material, such as we consider in our experiments and modeling, will generate an Oersted field that can be calculated by means of the Biot-Savart law, where

$$H^{\text{Oersted}}\left(x,t\right)=\frac{1}{4\pi }\int ‍j\left(x\prime ,t\right)\times \frac{\left(x-x\prime \right)}{{|x-x\prime |}^{3}}\;dx$$ (2)

where **j**(**x**′, *t*) is the space- and time-dependent current density flowing through the conductor. In our modeling, we assume that the current density is homogeneous and thus a spatially uniform and constant vector that is only time-dependent. This equation is solved in the Fourier space, similar to the calculation of the demagnetization field, using a fast Fourier transform algorithm, which provides a substantial speedup. For simulations where we consider the current-driven excitation of spin waves, the Oersted field is solved for each time step according to the adaptive time-stepped Runge-Kutta-Fehlberg time integrator provided as default in magnum.np. For the stripline antenna simulations, we precompute the Oersted field at a constant current density *j* = 1 × 10^9^ A m^−2^, and take the maximum of the *y* component of the calculated Oersted field as the value for the spatially homogeneous, stripline generated field.

### Spin-transfer torque

The spin of the electrons flowing through our magnetic stack can be polarized due to the magnetization. Such spin-polarized electrons will exert a torque on the magnetization, called the STT. This additional, current-induced torque can be modeled in a continuum theory by using the Zhang and Li approach (*54*), where the STT is modeled as an additional torque in the LLG equation mentioned above and hence becoming the Landau-Lifshitz-Gilbert-Slonczewski equation, where

$$\frac{\partial m}{\partial t}=-\gamma m\times H^{\text{eff}}+\alpha m\times \frac{\partial m}{\partial t}+T_{\text{STT}}$$ (3)

The additional torque

$$T_{\text{STT}}=-b\times m\times \left[m\times \left(j_{e}\cdot \nabla \right)m\right]-b\xi m\times \left(j_{e}\cdot \nabla \right)m$$ (4)

describes how the STT acts due to the local gradient of the magnetization, where ξ is the degree of nonadiabacity and *b* is described as

$$b=\frac{\beta \mu_{B}}{eM_{s}\left(1+\xi^{2}\right)}$$ (5)

The polarization rate of the conducting electrons, which have an elementary charge of *e*, is symbolized by β. The Bohr magneton is denoted by μ_B_. For all simulations involving the STT, we use ξ = 0.05 and *b* = 72.17 × 10^−12^.
