# Supplementary material for: Steerable current-driven emission of spin waves in magnetic vortex pairs
Source: Sci Adv. 2024 Sep 25;10(39):eado8635. doi: 10.1126/sciadv.ado8635 (PMC11423888; doi:10.1126/sciadv.ado8635)
Supplement: Supplementary file 1 — Figs. S1 to S15 Legends for movies S1 to S8 [file sciadv.ado8635_sm.pdf]

Science Advances
AAAS

Supplementary Materials for

Steerable current-driven emission of spin waves in magnetic vortex pairs

Sabri Koraltan *et al.*

Corresponding author: Sabri Koraltan, sabri.koraltan@univie.ac.at;
Sebastian Wintz, sebastian.wintz@helmholtz-berlin.de

Sci. Adv. **10**, eado8635 (2024)
DOI: 10.1126/sciadv.ado8635

The PDF file includes:

Figs. S1 to S15
Legends for movies S1 to S8

Other Supplementary Material for this manuscript includes the following:

Movies S1 to S8

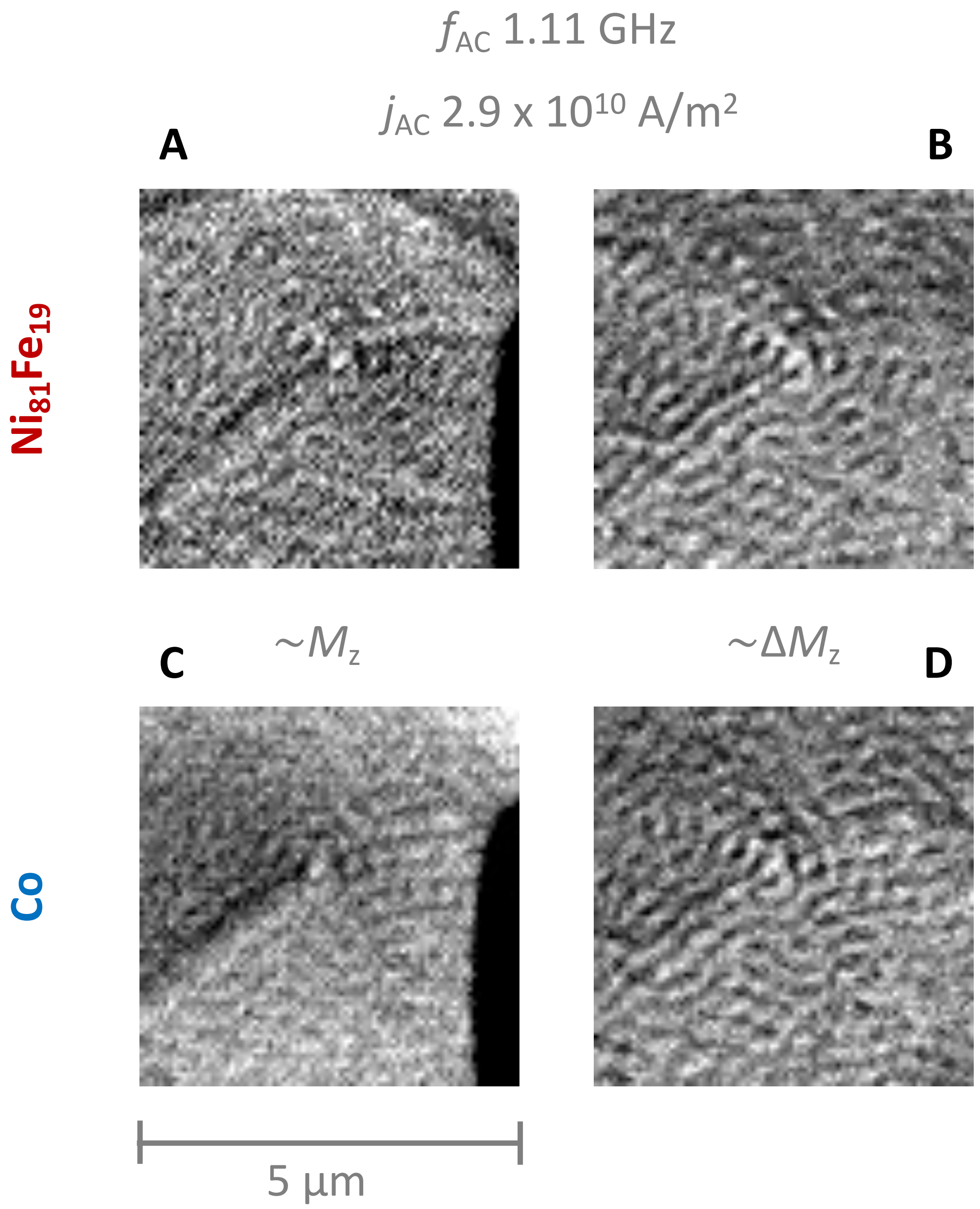

Figure S1: **Layer-specific TR-STXM imaging of current-induced spin-wave emission in SFi Sample #1.** Response to an alternating current of $f_{AC} = 1.11\,\text{GHz}$ and $j_{AC} = 2.9 \times 10^{10}\,\text{A/m}^2$ as in Fig. 2 of the main text. (A,C) direct absorption snapshots showing both topographic and magnetic contrast, the latter with perpendicular sensitivity ($\sim M_z$). (B,D) normalized snapshots highlighting the magnetic dynamics ($\sim \Delta M_z$). Images recorded at the Ni L_3 edge (A,B) (representative for the top layer) and the Co L_3 edge (C,D) (representative for the bottom layer), respectively. These images provide futher evidence for the local interlayer coupling of the spin textures (matching domain wall signatures in A,C), the parallel orientation of the vortex cores (central white dots in A,C), and layer-collective spin-wave dynamics (congruent wave patterns in B,D).

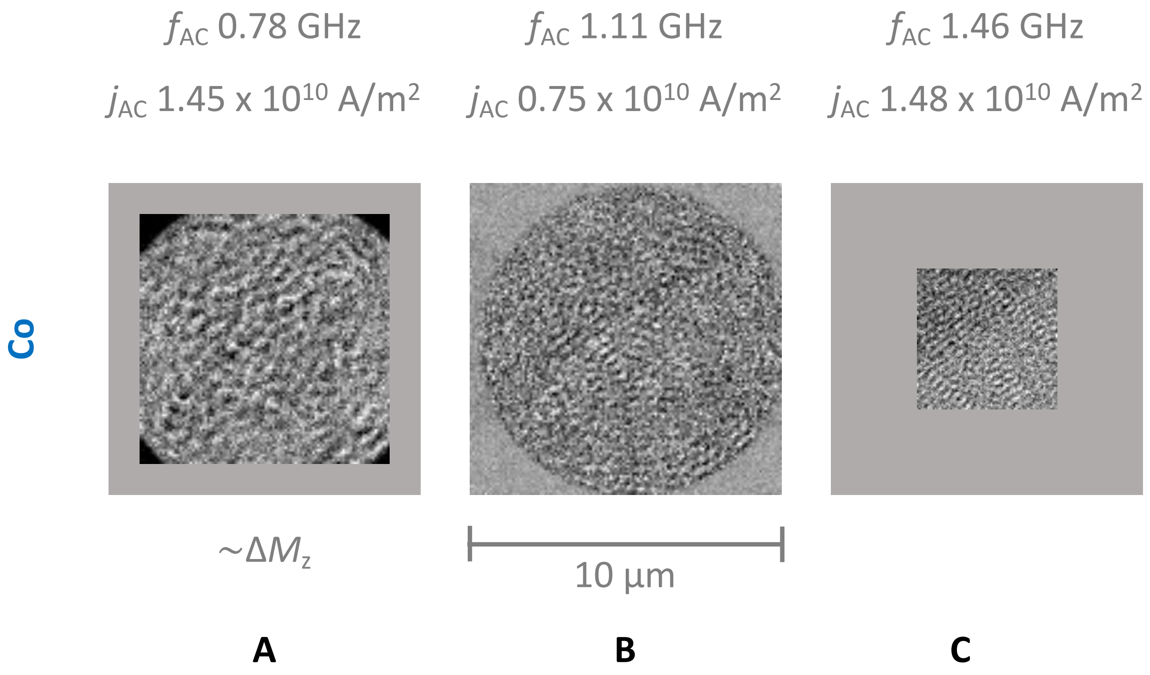

Figure S2: **TR-STXM imaging of current-induced spin-wave emission in SFi Sample #1 at different frequencies.** Response to alternating currents of (A) $f_{AC} = 0.78\,$GHz, (B) $f_{AC} = 1.11\,$GHz, and (C) $f_{AC} = 1.46\,$GHz, with current densities as given. Snapshot images recorded at the Co L_3 edge with normalized contrast that highlights magnetic dynamics ($\sim \Delta M_z$). The emission of spin waves is present at all three different frequencies, underlining the versatility of the broadband excitation process. As expected (*25*), the emitted spin-wave wavelengths decrease with increasing frequency, from 533 ± 30 nm at 0.78 GHz, via 320 ± 15 nm at 1.11 GHz, down to 250 ± 15 nm at 1.46 GHz.

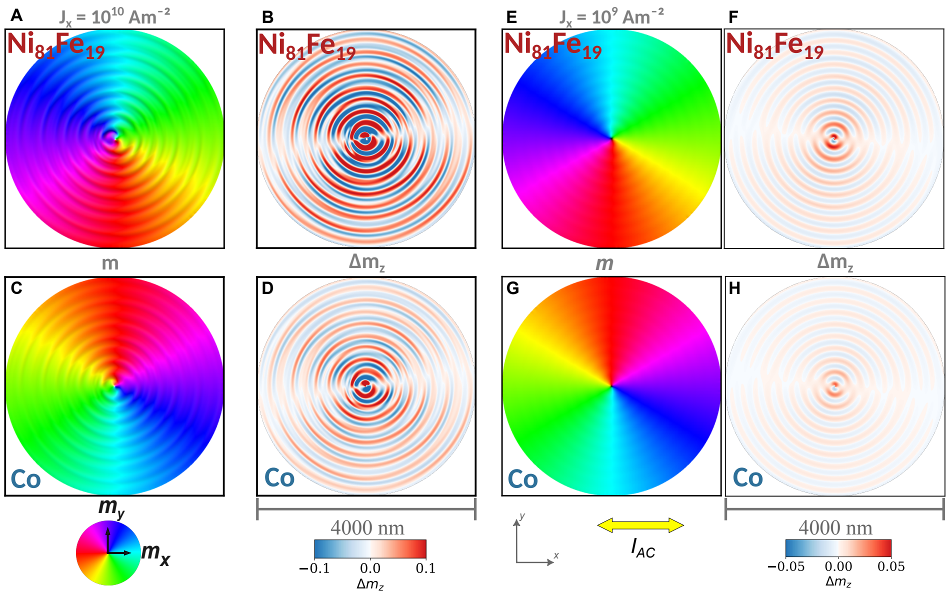

Figure S3: **Layer-resolved current-induced spin-wave emission in SFi vortex pairs (simulations).** In-plane components highlighted with a color wheel, where the excitation of spin waves is slightly visible in the darker shades of A ($Ni_{81}Fe_{19}$) and C (Co) for an a current density amplitude of $j = 1 \times 10^9\,\mathrm{A/m^2}$. In B ($Ni_{81}Fe_{19}$) and D (Co) we highlight the spin-wave pattern by depicting the change in the z component of the magnetization Δm_z. In E to H, we show corresponding images for an increased current amplitude of $j = 1 \times 10^{10}\,\mathrm{A/m^2}$, leading to substantial increase of the spin-wave amplitude at a simultaneous occurrence of a slight double-frequency ($2f$) component in the spin-wave response.

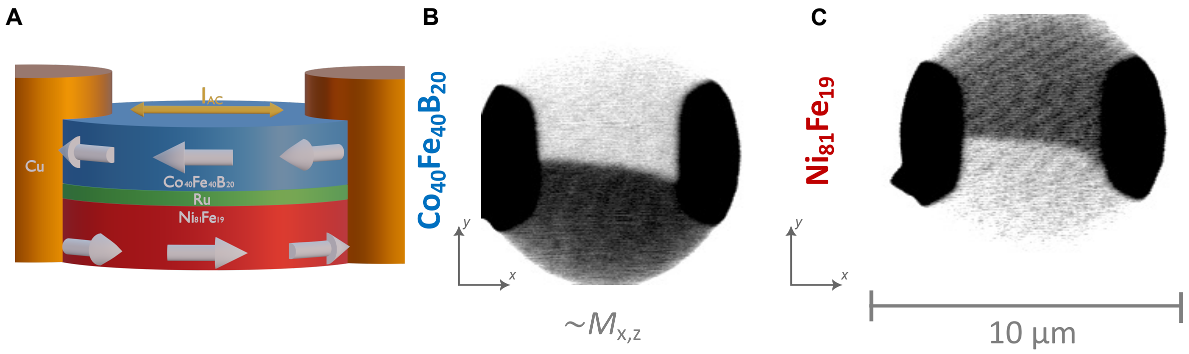

Figure S4: **Sample schematic and STXM imaging of SFi Sample #2.** (A) 3D schematic of the $Ni_{81}Fe_{19}/Ru/Co_{40}Fe_{40}B_{20}$ microdisk with adjacent, partially overlapping copper leads (aspect ratios not to scale). White arrows indicate the general antiparallel orientation of the two ferromagnetic layers of the SFi. Alternating current injected from the leads flow laterally through the disk as supported by the orange arrow. Static STXM images with topographic and partial in-plane magnetic sensitivity ($\sim M_{x,z}$), recorded at the Co L_3 edge (B) and the Ni L_3 edge (C), respectively. The STXM images confirm antiparallel alignment of the in-plane magnetic components of the two ferromagnetic layers through antiferromagnetic interlayer exchange coupling.

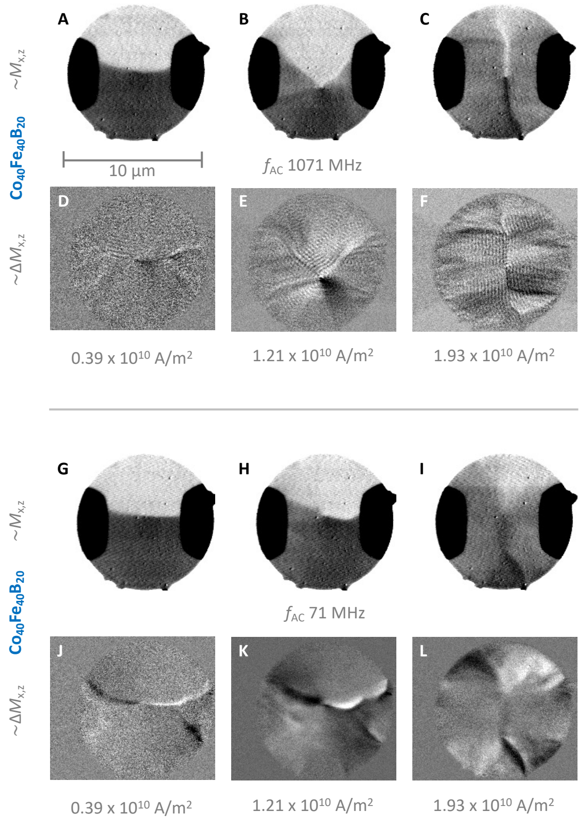

Figure S5: **TR-STXM imaging of direction-steerable spin dynamics in SFi Sample #2 at different frequencies.** Response to alternating currents of $f_{AC} = 1071\,\text{MHz}$ (A-F) and $f_{AC} = 71\,\text{MHz}$ (G-L) with current densities as provided below the individual columns. Images recorded at the Co L_3 edge. Rows (A-C and G-I), direct absorption snapshots showing both topographic and magnetic contrast, the latter with mixed sensitivity ($\sim M_{x,z}$). Rows (D-F and J-L), normalized snapshots highlighting the magnetic dynamics ($\sim \Delta M_{x,z}$). The domain walls reorient from horizontal to vertical in the micrographs with increasing current densities. At $f_{AC} = 1071\,\text{MHz}$ this leads to a change of the (directional) spin-wave emission pattern from horizontal plane waves, via somewhat isotropic emission, to vertical plane waves. As expected, the average wavelength of 263 ± 15 nm is shorter than the wavelengths in the $f_{AC} = 571\,\text{MHz}$ case shown in Fig. 6 of the main text. At $f_{AC} = 71\,\text{MHz}$ the domain walls oscillate, however, spin waves are not noticeably excited, in line with a finite spin-wave frequency gap of the order of 500 MHz (*42*).

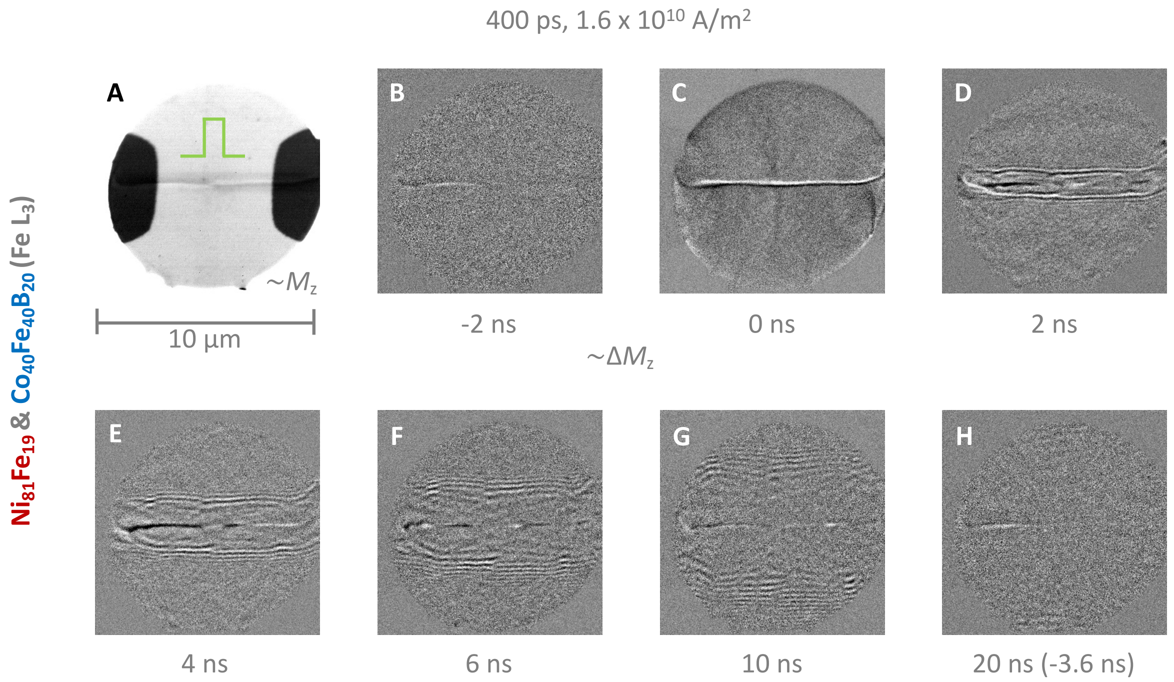

Figure S6: **TR-STXM imaging of pulsed current-induced spin-wave dynamics in Sample #2.** Response to a current pulse of 400 ps duration and $1.6 \times 10^{10} \mathrm{A/m^2}$ amplitude. Images recorded at the Fe L_3 edge, with sensitivity to the magnetization of both ferromagnetic layers. (A) Static STXM image with topographic and perpendicular magnetic contrast ($\sim M_z$). (B-H) TR-STXM normalized snapshots at different delay times, sensitive to the perpendicular magnetic dynamics ($\sim \Delta M_z$). The total stroboscopic obeservation time is 23.6 ns. (B) Situation 2 ns before the pulse, (C) at the time of the pulse, (D) 2 ns, (E) 4 ns, (F) 6 ns, (G) 10 ns after the pulse. (H) 20 ns after (or 3.6 ns before) the pulse. According to the micrographs, the current pulse generates a spin-wave wave-packet, emitted from the domain walls. The dynamics highlight the agility and non-resonant character of the excitation process.

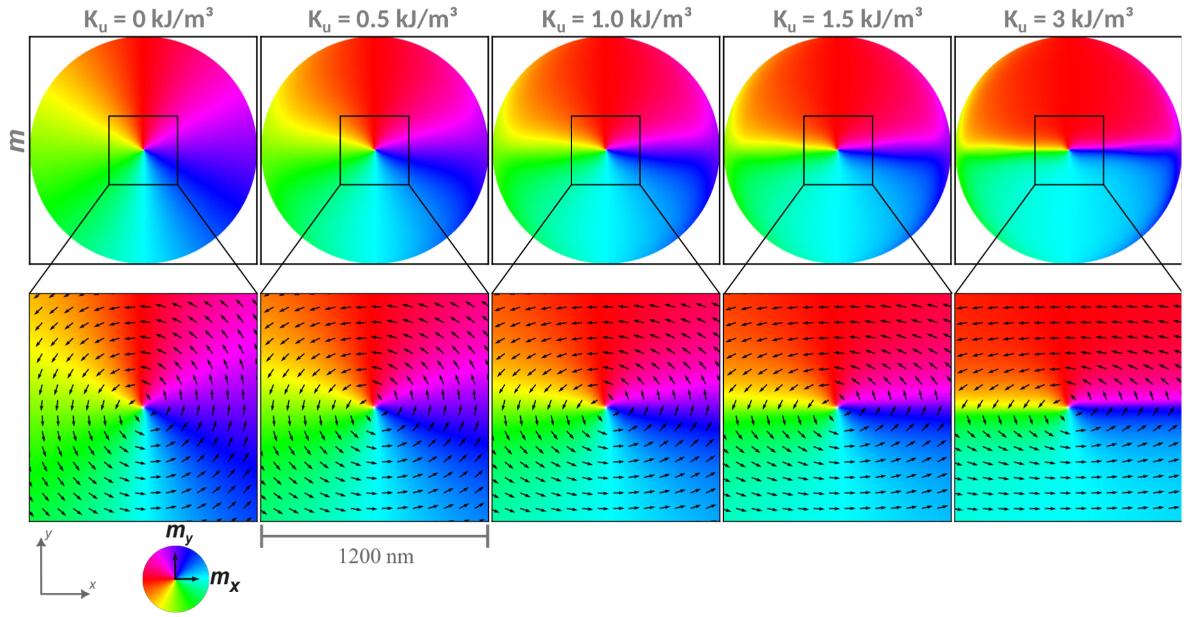

Figure S7: **Influence of additional uniaxial anisotropy in the CoFeB layer (simulations).** The magnitude of the uniaxial anisotropy in $Co_{40}Fe_{40}B_{20}$ is varied. The color-coded figures illustrate the in-plane component of the magnetization, while the images below focus on a smaller area around the vortex core, with black arrows indicating the direction of the magnetization. It is evident that the vortex state is stretched into a more parallel domain state as K_u is increased.

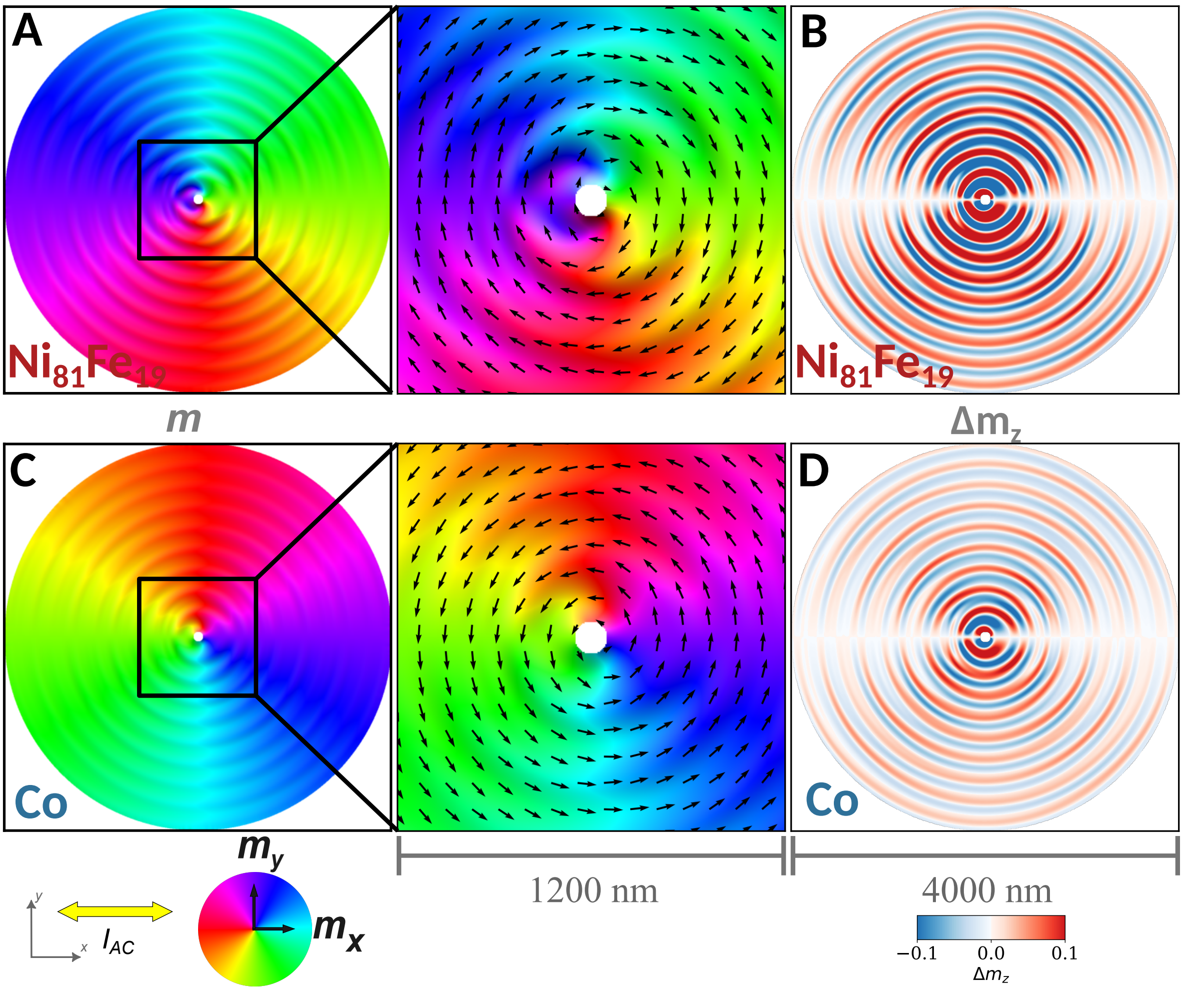

Figure S8: **Impact of artificially removed vortex cores on the spin-wave emission (simulations)**. If the cores are removed in the simulations, the current-driven Oersted excitation still leads to an efficient generation of spin waves. In A (C) we show the in-plane components of the magnetization of the $Ni_{81}Fe_{19}$ (Co) layer, where the black box zooms into the area around the missing vortex core. The excitation of spin waves is already visible in this view. The corresponding perpendicular dynamic magnetization component ΔM_z is depicted in B and D, respectively.

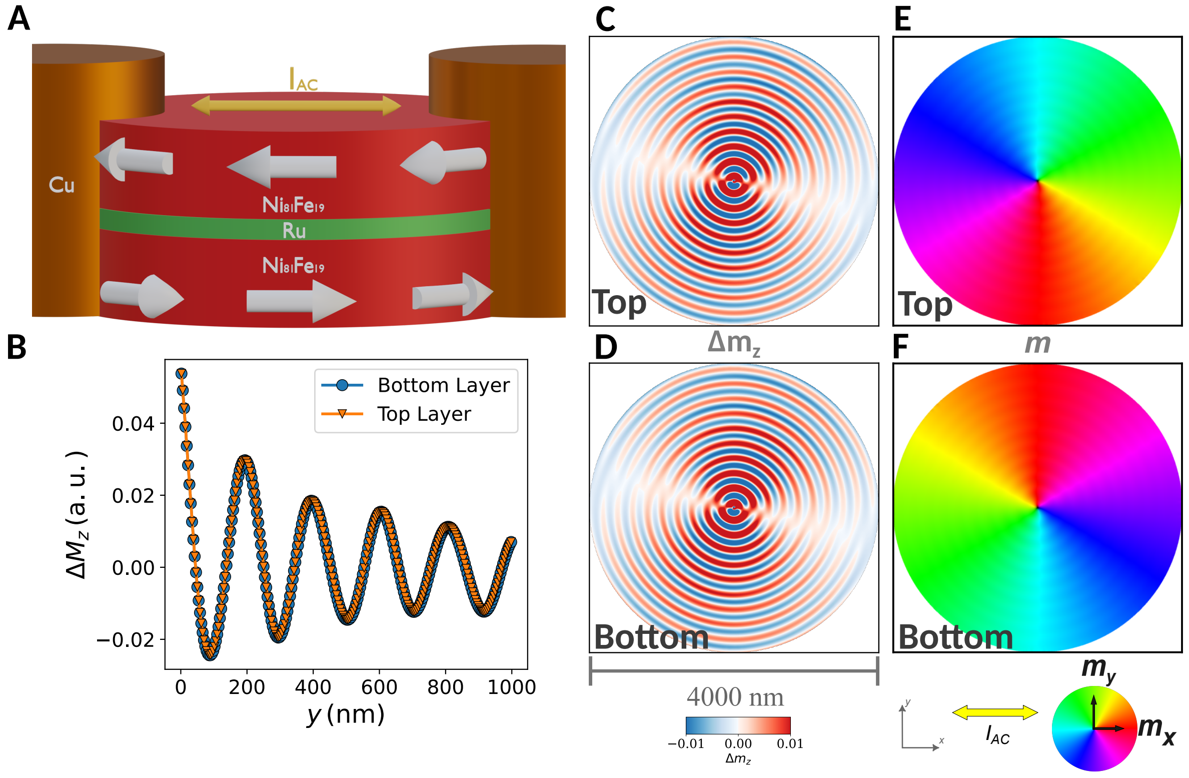

Figure S9: **Spin waves in fully compensated SAFs (simulations).** 3D representation of the compensated SAF geometry in (A) where two NiFe layers are coupled through a thin Ru layer. The Oersted field driven generation of spin waves is simulated with the same NiFe material parameters as for Sample #1. The current density is $j_x = 10^9\,\mathrm{A/m^2}$ and the excitation frequency is $f = 1.11\,\mathrm{GHz}$. Line plot of ΔM_z is given in (B), with the corresponding snapshots of Δm_z and color coded magnetization in (C) and (E) for the top layer, and in (D) and (F) for the bottom layer. The overlap of the two curves in (B) is a strong index for the in-phase excitation of the spin waves in the two layers that are perfectly compensating the total magentization.

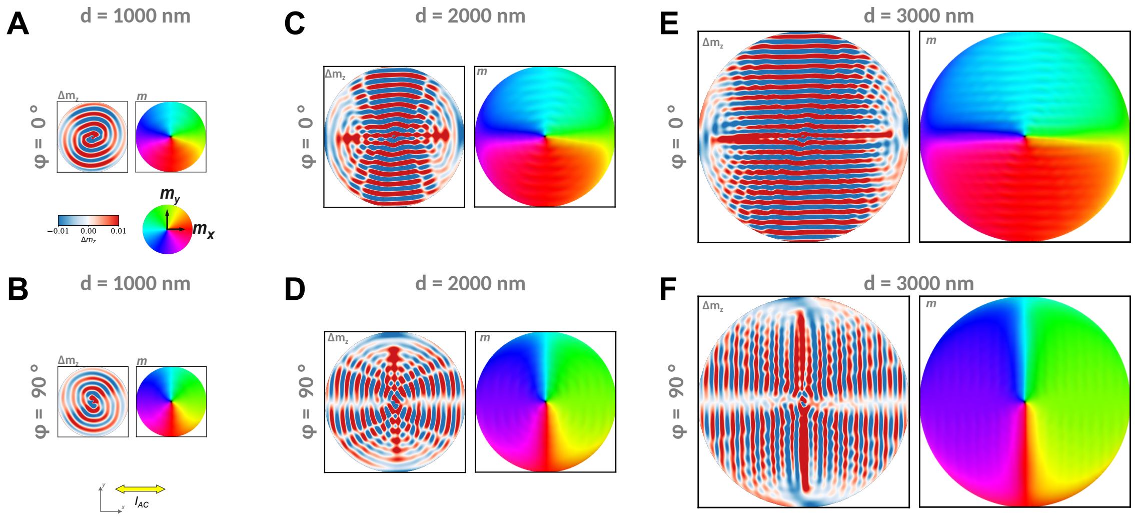

Figure S10: **Influence of geometrical constraints on the steering of spin waves (simulations).** Sample #2 was simulated using different disk diameters of 1 μm (A,B), 2 μm (C,D) and 3 μm (E,F), for the two orthogonal anisotropy directions $\varphi = 0°$ (A,C,E) and $\varphi = 90°$ (B,D,F). While plane waves can be obtained in a more defined pattern in larger disks, the steering of spin-waves also works for smaller disks, as illustrated by the snapshots of the change of the magnetization Δm_z and the colorwheel representations of magnetization.

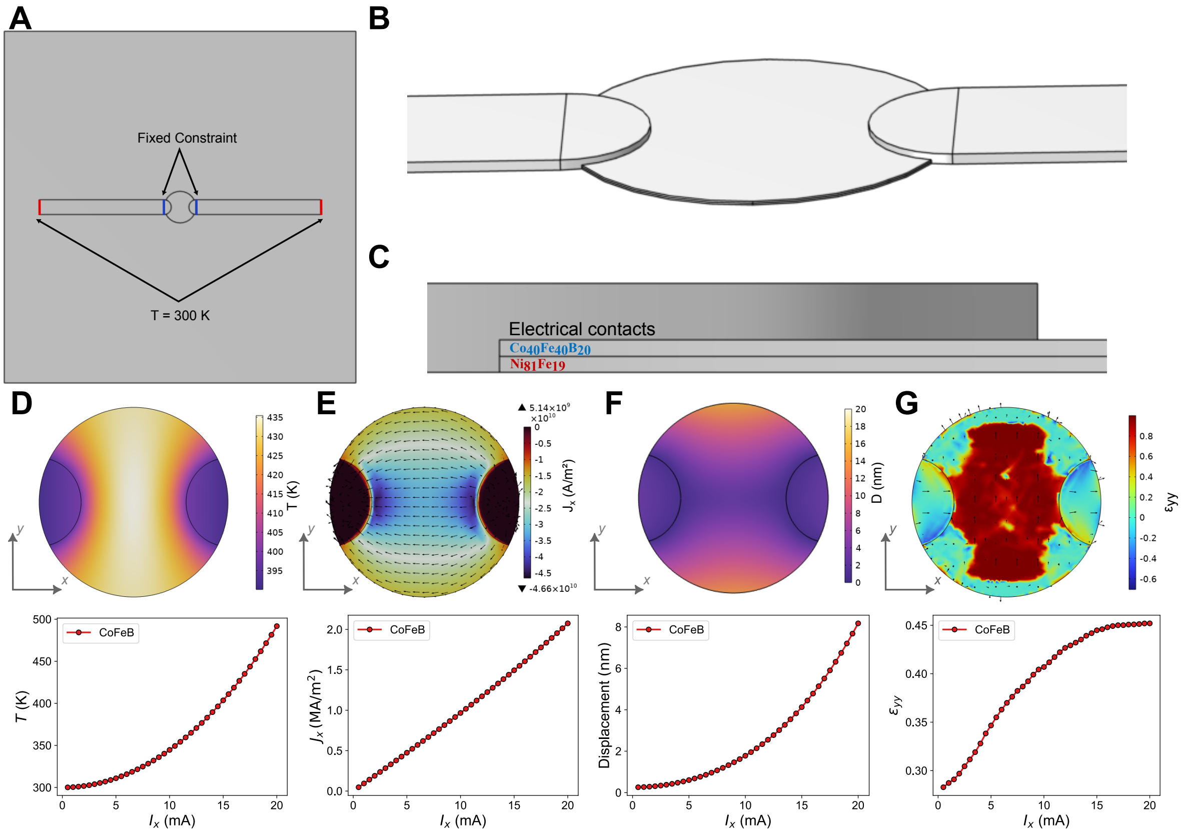

Figure S11: **Summary of Comsol Simulations.** Top view of the simulated structure in (A) and zoomed side views in (B) and (C), where it can be seen how the contacting is realized on the two layers of interest. The boundary conditions used for temperature and geometrical constraints are highlighted in (A). We simulate only the CoFeB and NiFe layers while omitting the Ru layer as it is very thin and hence not expected to significantly change the results. This simplification led to much shorter computation times due to a larger finite-element discretization. The input current is varied and we plot the dependence of the Joule heating (D), current density (E), total displacement magnitude (F) and the y component of the strain along y (G). The snapshots of the state in the upper panels are taken at $I_x = 16\,\mathrm{mA}$. From F and G it can be understood how the heating of the sample leads to a strain which in turn gives rise to an anisotropy that allows the steering of spin waves. In the COMSOL simulations, we model the disks with their experimental thicknesses as well as lateral dimensions. A 200 nm thick SiN membrane has been used to mimic the membrane required for STXM imaging. To couple the effects of electrical current flow, Joule heating and mechanical deformations, we include the electric currents, heat transfer in solids, and solid mechanics modules into the multiphysics software. The lateral contacts are assumed to be made of pure copper. For the NiFe layer we chose the HyMu 80 alloy (Permalloy) as the closest material to our system. For the CoFeB we started from a polycrystalline solide cobalt and changed certain material parameters based on the literature, such as: Young's modulus $E_F = 200\,\mathrm{GPa}$, Poisson's ratio $\nu = 0.3$, and the thermal conductivity $k_{\mathrm{iso}} = 69.21\,\mathrm{W/(m \cdot K)}$.

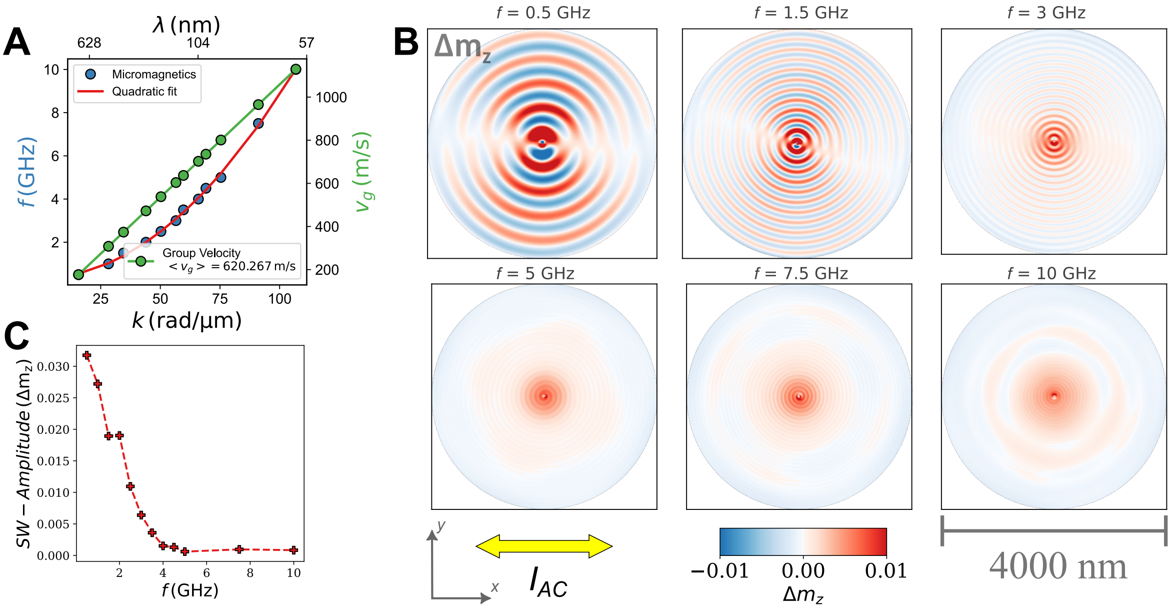

Figure S12: **Dispersion relation for Sample #1 (simulations).** The dispersion relation obtained via micromagnetic simulations is given in (A) with the spin waves being excited by the Oersted field. The results demonstrate the broadband functionality of the proposed excitation mechanism. The extracted data points (blue) are fitted by a quadratic function (red line) and from the slope of which, the group velocities (green) are computed. An average group velocity of $< v_g >= 620\,\mathrm{m/s}$ is obtained. In the simulations, wavelengths as low as 60 nm can be achieved by increasing the excitation frequency accordingly. In (B) we provide snapshots of the dynamic component Δm_z of $Ni_{81}Fe_{19}$ with the same color range for all figures to highlight the reduction in the spin-wave amplitude with increasing frequency, as well as the decrease in wavelength. In (C) we quantified the amplitude of the spin waves as a function of excitation frequency by taking into account the average spin-wave oscillation amplitude 500 nm away from the vortex core.

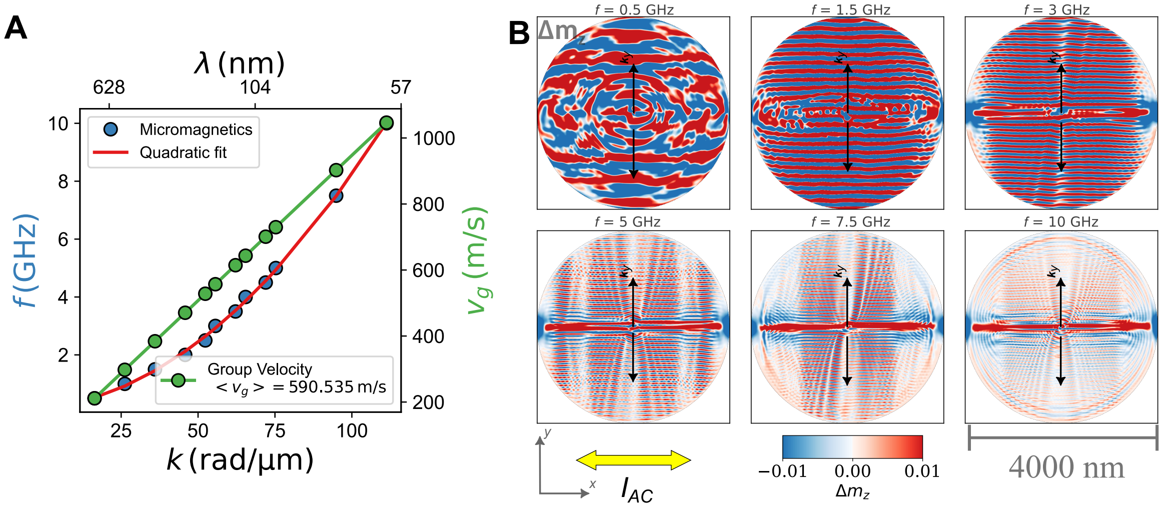

Figure S13: **Dispersion relation for Sample #2 and $\varphi = 0^\circ$ (simulations).** Same as Supplementary Fig. S12 where it applies. The orientation of the uniaxial anisotropy axis is set such that $\varphi = 0^\circ$ (horizontal axis).

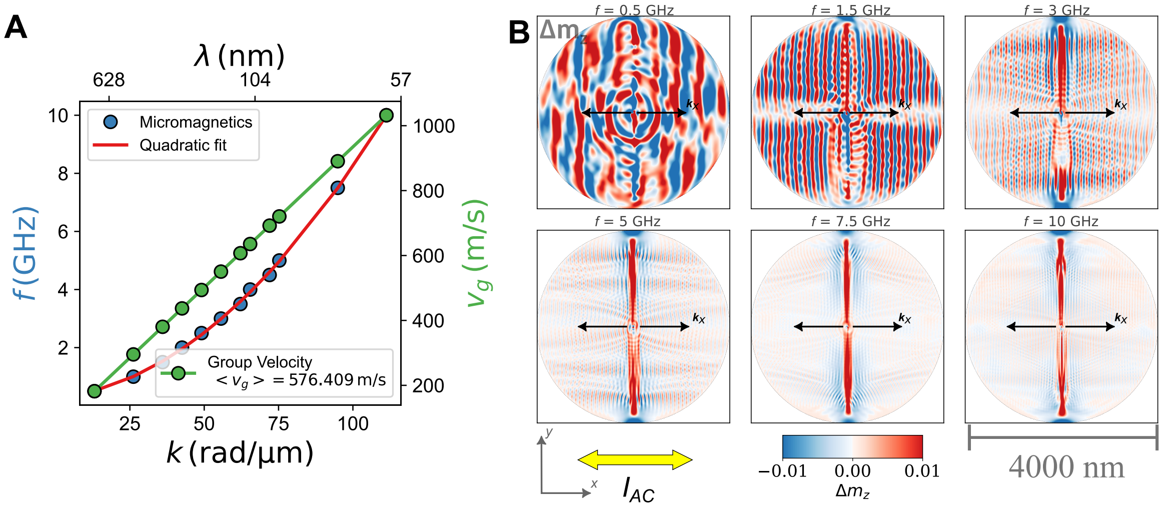

Figure S14: **Dispersion relation for Sample #2 and $\varphi = 90^\circ$ (simulations).** Same as Supplementary Fig. S13 but with the orientation of the uniaxial anisotropy axis set such that $\varphi = 90^\circ$ (vertical axis).

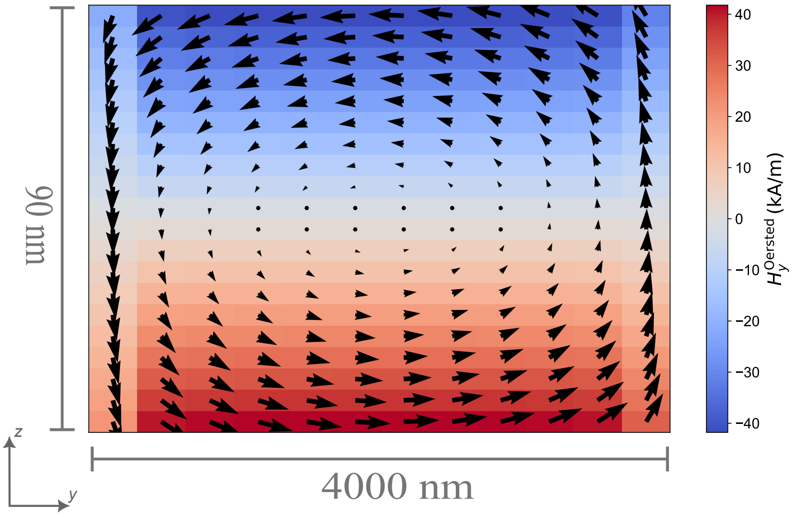

Figure S15: **Oersted field profile (simulations).** Cross-section profile of the calculated Oersted Field from micromagnetic simulations to highlight the field distribution in the $yz-$plane (aspect ratio not to scale).

Movie S1. Experimental results from the spin-wave dynamics in Sample #1 which are layer resolved. Both absolute and normalized spin-wave amplitudes are given.

Movie S2. Experimental results from the spin-wave dynamics in Sample #1 for different frequencies, where the normalized amplitude is illustrated.

Movie S3. Simulations using the parameters of Sample #1, where we provide the absolute and normalized illustrations of the spin-wave excitations layer resolved for both layers.

Movie S4. Simulations using the parameters of Sample #1 where different excitation methods are compared.

Movie S5. Experiments on Sample #2 which show the time-resolved dynamics analogous to statics presented Figure 6

Movie S6. Experiments on Sample #2 which show the time-resolved dynamics analogous to statics presented Figure S5

Movie S7. Simulations using the material parameters of Sample #2 where we show the time resolved dynamics analogous to Figure 7.

Movie S8. Experiments on Sample #2 which show the time-resolved dynamics analogous to Figure S7.
